# The application of pre-trained large visual-language models for preliminary diagnosis of esophageal whitish plaques in large-scale esophageal cancer screening

**DOI:** 10.1038/s41698-026-01301-8

**Published:** 2026-01-28

**Authors:** Yilin Li, Xin Li, Di Zhang, Wenwen Zhu, Yuan Hu, Zijian Zhao, Qi Zhao

**Affiliations:** 1 School of Control Science and Engineering, Shandong University, Jinan, Shandong Province China; 2 Department of Gastroenterology, Shandong Provincial Hospital Affiliated to Shandong First Medical University, Jinan, Shandong Province China; 3 Medical examination center and electrocardiogram room, Shandong Acupuncture and Moxibustion Hospital Affiliated to Shandong Academy of Traditional Chinese Medicine, Jinan, Shandong Province China; 4 Department of Gastroenterology, Shandong Provincial Hospital, Shandong University, Jinan, Shandong Province China; 5 Shandong Booke Biotechnology Co. Ltd., Jinan, Shandong Province China

**Keywords:** Cancer, Gastrointestinal cancer

## Abstract

Esophageal whitish plaques are common findings in large-scale esophageal cancer screenings, requiring accurate preliminary differentiation to guide appropriate clinical management. This study presents a computer-aided diagnosis (CAD) system based on the pre-trained large-scale visual-language (VL) model BLIP for automated diagnosis and description of esophageal whitish plaques. A dataset of 13,922 endoscopic images was used for model training, and comparative experiments were conducted with multiple benchmark models, including Poolformer, Swin-Transformer, TransMSF, and ViT. The results demonstrate that our approach outperforms existing methods in terms of precision, recall, F1 score, and accuracy. Compared with LLaVA-Med, our model significantly improves keyword accuracy (K-ACC) in medical text descriptions. A human-machine competition further demonstrated that our model outperforms both senior and junior endoscopists, particularly excelling in the recall of early esophageal cancer cases. These findings suggest that integrating pre-trained VL models into CAD systems can enhance the accuracy and efficiency of esophageal whitish plaque diagnosis, reducing misdiagnoses and supporting clinical decision-making.

## Introduction

The incidence of digestive diseases remains alarmingly high worldwide, posing a significant threat to human health, particularly in the form of digestive tract cancers, especially esophageal cancer, which claims the lives of 1.8 million people annually^1^. Digestive endoscopy plays a pivotal role in the early detection of gastrointestinal diseases, particularly in identifying early-stage gastrointestinal malignancies, enabling timely intervention and achieving nearly 100% survival rates^2^. In recent years, China has been actively promoting the adoption of these examinations in primary healthcare facilities and conducting large-scale screening campaigns annually to enhance early diagnosis and treatment outcomes.

**Fig. 1:**
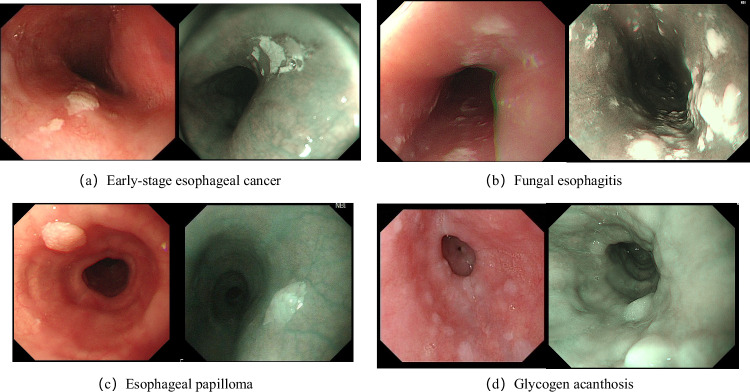
Representative endoscopic images of four esophageal diseases. White-light endoscopy (WLE) images are presented on the left and narrow-band imaging (NBI) images are presented on the right. All images illustrate characteristic whitish plaques. Panels are arranged as follows: (**a**) Early-stage esophageal cancer (upper left); (**b**) Fungal esophagitis (upper right); (**c**) Esophageal papilloma (lower left); (**d**) Glycogen acanthosis (lower right).

In large-scale esophageal cancer screening, esophageal whitish plaques are very common^3,4^. In clinical practice, endoscopic whitish plaques in the esophagus do not represent a single disease diagnosis. Instead, they refer to whitish visual changes on the surface of the esophageal mucosa that have a clear boundary with the surrounding normal pink mucosa. The causes of esophageal whitish plaques are numerous. According to our clinical screening statistics, over 20% of patients undergoing gastroscopy will exhibit esophageal whitish plaques that are difficult to erase. Among these cases, a significant portion of the whitish plaques is caused by tightly adherent inflammatory materials, which could be inflammatory exudates on the surface of early esophageal cancer^5^, or plaques and inflammatory deposits in fungal esophagitis^6,7^. The remaining cases may be due to excessive or uneven keratinization of the squamous epithelium, manifesting as glycogenic acanthosis^8,9^ or esophageal papilloma^10^. Specifically, the representative endoscopic appearances of the four diseases included in Fig. 1 are as follows:

- Early-stage esophageal cancer: Presents as plaque-like whitish mucosal areas with slightly elevated and rough surfaces. These white regions result from reduced blood supply or surface exudation and cannot be removed by wiping.
- Fungal esophagitis: Shows punctate, patchy, or geographic white pseudomembranes with a soft ”tofu-dreg-like” texture. These pseudomembranes may detach when flushed or gently scraped, revealing hyperemic or eroded mucosa underneath.
- Esophageal papilloma: Appears as small, white, papillary or granular protrusions. These lesions are usually lobulated and sessile or sub-pedunculated. Under magnified narrow-band imaging, vascular changes can be observed, but without atypical features.
- Glycogen acanthosis: Characterized by multiple small, flat white elevations with sharp boundaries and a slightly granular appearance. Under magnification, the vascular texture disappears. This benign lesion can be distinguished from early cancer using iodine staining.

For these four scenarios, endoscopists need to perform critical preliminary differential diagnoses based on endoscopic images and take different actions accordingly. If a preliminary diagnosis suspects early esophageal cancer, a key biopsy should be promptly conducted. For cases preliminarily diagnosed as fungal esophagitis, a smear biopsy or direct recommendation for antimicrobial therapy is required. Patients preliminarily diagnosed with glycogenic acanthosis or esophageal papilloma may not need an immediate biopsy but can be managed through follow-up observation. Diagnosis relies heavily on the physician’s experience and skill in interpreting subtle differences in endoscopic images. This leads to significant inter-physician variability and a higher risk of misdiagnosis.

Given the over-reliance on doctors’ capabilities and experience for the clinical preliminary diagnosis of esophageal whitish plaques, diagnostic accuracy varies, with more experienced senior diagnostic physicians performing better, followed by those with average years of experience, and the worst being newly graduated medical students. Considering the widespread application of artificial intelligence (AI)^11–14^, especially convolutional neural network (CNN) and large Visual-Language (VL) models, in the medical field, proposing an AI system to assist doctors in completing the preliminary diagnostic tasks for esophageal whitish plaques could significantly improve efficiency and accuracy. Therefore, this paper presents a computer-aided diagnostic (CAD) system based on the pre-trained large VL model—BLIP^15^ for the preliminary differential diagnosis of esophageal whitish plaque images. Compared with traditional CAD methods for endoscopic images^13,14^, the proposed approach based on the VL model not only provides diagnostic outcomes but also generates textual descriptions of endoscopic images, which represents a significant advantage unmatched by conventional techniques.

## Results

### Experiment Settings

The proposed model is implemented on the Ubuntu 22.04 LTS operating system using the PyTorch 2.0 framework based on Python 3.10, CUDA 11.8, and CUDNN 7.4. The Tesla V100 32G GPU is used as an accelerator for training. The training model initializes the learning rate to 5 × 10^−5^ and uses the momentum method to train. The momentum parameter is set to 0.9, the weight decay is set to 0.0005, the learning rate decay step is 5, the batch size is set to 16, and the training epoch is set to 100.

### Comparison with other CNN models

To evaluate the diagnostic performance of the proposed model, we compared it with 4 state-of-the-art (SOTA) CNN models, namely, Poolformer^16^, Swin-transformer^17^, TransMSF^18^, ViT^19^. We trained these 4 CNN models using the data from the Training Set. Subsequently, we tested each model using the Validation Set and calculated four metrics for each model: Precision (PREC), Recall (REC), F1 score, and Accuracy (ACC), as shown in Table 1 (PREC, REC, and F1 score are the average values of the four classes). The experimental results in Table 1 show that, under both white-light endoscopy (WLE) and narrow-band imaging (NBI) modalities, the proposed model has the highest performance indicators in classifying the four digestive tract diseases compared with the four state-of-the-art CNN models.

**Table 1:** Comparison of all models’ performance indicators: Precision (PREC), Recall (REC), F1 score, and Accuracy (ACC)

Model	WLE				NBI			
	PREC	REC	F1	ACC	PREC	REC	F1	ACC
Poolformer	0.8171	0.7944	0.8027	0.8153	0.7422	0.7272	0.7344	0.7468
Swin-transformer	0.8405	0.8237	0.8304	0.8382	0.7902	0.7742	0.7799	0.7945
TransMSF	0.8687	0.8434	0.8406	0.8652	0.8442	0.8158	0.8266	0.8408
ViT	0.8271	0.8074	0.8151	0.8266	0.7309	0.7023	0.7117	0.735
**Ours**	**0.8739**	**0.8808**	**0.8766**	**0.8905**	**0.8630**	**0.8673**	**0.8644**	**0.8711**

PREC, REC and F1 score are the average values of the four classes.

Bold values denote the highest performance achieved among the compared methods.

### Comparison with other VL models

To evaluate the performance of the proposed model in image-text description tasks, we compared it with LLaVA-Med^20–22^, a dedicated VL model pre-trained on a biomedical dataset. Specifically, LLaVA-Med was trained using the publicly available PMC-5M dataset^22^, which comprises 15 million biomedical image-text pairs across diverse categories, including medical images and corresponding textual annotations such as captions or diagnostic reports. We also conducted instruction fine-tuning training on the LLaVA-Med model by using the Training Set.

The performance of both models was assessed using the Keyword Accuracy (K-ACC) metric. We defined four medical keywords: ”fungal esophagitis”, ”esophageal cancer”, ”esophageal papilloma”, and ”glycogenic acanthosis”. A prediction was marked correct if the VL model’s generated description of an image contained at least one keyword that matched the ground-truth annotation for that image (Images with mismatched classification and keywords were excluded from K-ACC calculation). The final K-ACC score was calculated as the ratio of correct predictions to the total number of test images. As shown in Table 2, the proposed model outperformed LLaVA-Med (original and fine-tuned) in overall K-ACC (the performance of the fine-tuned LLaVA-Med model is much better than the original LLaVA-Med model’s, the overall K-ACC has increased by 30% compared to that before fine-tuning). For disease-specific K-ACC, the fine-tuned LLaVA-Med model outperformed the proposed model only in Glycogenic Acanthosis. For the LLaVA-Med model in the diagnosis of WLE and NBI endoscopic images, especially in the diagnosis of WLE-type endoscopic images, the K-ACC is relatively low. In contrast, the K-ACC of the model proposed in this paper is relatively high (more than 37% at least). This demonstrates its superiority in accurately identifying and describing key pathological features in biomedical images. The evaluation methodology ensured a rigorous comparison by focusing on clinically relevant keywords, aligning with the diagnostic requirements of medical practitioners.

**Table 2:** Experiment results in the keyword accuracy (K-ACC) for LLaVA-Med and our model

Category	LLaVA-Med		Ours
	Original	Fine-tuned	
Overall	0.3462	0.6460	0.8480
Fungal Esophagitis	0.2355	0.6000	0.9026
Esophageal Cancer	0.4831	0.7536	0.8966
Esophageal Papilloma	0.4001	0.6480	0.8129
Glycogenic Acanthosis	0.1579	0.8289	0.7645
WLE	0.2583	0.4768	0.8534
NBI	0.5091	0.6667	0.8425

### Human-machine reading competition

To compare the diagnostic performance between the model proposed in this paper and endoscopists, we organized a human-machine reading competition. Four endoscopists participated in the competition, including two senior ones with 10–20 years of experience and two junior ones with 5–10 years of experience. A total of 596 images used in the competition (298 images each for WLE and NBI) were randomly selected from the test set. During the competition, each endoscopist only needed to determine the disease type of the images. By comparing their judgments with the annotated diagnostic results of the images, a confusion matrix could be obtained for each endoscopist. By aggregating all the confusion matrices, the experimental results for the metrics of PREC, REC, F1 score, and ACC could be obtained, as shown in Table 3.

**Table 3:** Results of the human-machine reading competition

		WLE				NBI			
		PREC	REC	F1	ACC	PREC	REC	F1	ACC
Senior1	EC	0.8734	0.9200	0.8961	0.8691	0.8551	0.7867	0.8194	0.8121
	FE	0.9875	0.9405	0.9634		0.8684	0.8148	0.8408	
	GA	0.7245	0.9467	0.8208		0.6569	0.8933	0.7571	
	EP	0.9756	0.6250	0.7619		0.9804	0.7463	0.8475	
Senior2	EC	0.9608	0.6533	0.7778	0.8624	0.9474	0.7200	0.8182	0.8557
	FE	0.8737	0.9881	0.9274		0.9036	0.9259	0.9146	
	GA	0.7347	0.9600	0.8324		0.7087	0.9733	0.8202	
	EP	0.9815	0.8281	0.8983		0.9636	0.7910	0.8689	
Junior1	EC	0.9302	0.5333	0.6780	0.7651	0.9750	0.5200	0.6783	0.7987
	FE	0.7788	0.9643	0.8617		0.6870	0.9753	0.8064	
	GA	0.6207	0.9600	0.7539		0.7556	0.9067	0.8242	
	EP	0.9800	0.5469	0.7071		0.9811	0.7761	0.8667	
Junior2	EC	0.8519	0.6133	0.7132	0.7752	0.7742	0.6400	0.7007	0.7685
	FE	0.7822	0.9405	0.8541		0.7895	0.9259	0.8523	
	GA	0.6535	0.8800	0.7500		0.6786	0.7600	0.7170	
	EP	0.9524	0.6250	0.7547		0.8596	0.7313	0.7903	
Ours	EC	0.8333	0.9244	0.8765	0.8905	0.9434	0.9259	0.9346	0.8696
	FE	0.9554	0.9414	0.9483		0.9006	0.9261	0.9132	
	GA	0.8129	0.7635	0.7875		0.8299	0.7673	0.7974	
	EP	0.8941	0.8941	0.8941		0.7731	0.8440	0.8070	

*EC* esophageal cancer, *FE* fungal esophagitis, *GA* glycogenic acanthosis, *EP* esophageal papilloma.

As shown in Table 3, regarding the WLE images, the senior endoscopists achieved ACC values of 86.91% and 86.24%, while the junior endoscopists achieved values of 76.51% and 77.52%. In contrast, the model achieved a high ACC of 89.05%. For NBI images, the ACC values were 81.21% and 85.57% for seniors, 79.87% and 76.85% for juniors. The model outperformed them, achieving an ACC of 86.96%. Endoscopists generally showed low recall in detecting early esophageal cancer. In WLE, the endoscopists achieved REC rates of 92.00%, 65.33%, 53.33%, and 61.33%, while in NBI, the rates were 78.67%, 72.00%, 52.00%, and 64.00%. However, the model exhibited significantly higher REC rates for diagnosing esophageal cancer, reaching 92.44% in WLE and 92.59% in NBI. These results revealed the superior performance of the model compared to the endoscopists.

## Discussion

In this study, we utilized the pre-trained VL model-BLIP to develop a novel model for the differential diagnosis of esophageal whitish plaques using two different imaging modalities, WLE and NBI. Compared with other SOTA CNN models (see Table 1), the model described in this paper exhibits the best performance in four metrics: PREC, REC, F1 score, and ACC. For WLE, it outperforms by at least 0.52%, 3.74%, 3.6%, and 2.53%, respectively; for NBI, it outperforms by at least 1.88%, 5.15%, 3.78%, and 3.03,% respectively. When compared with the LLaVA-Med (original and fine-tuned) in terms of the performance of text description output for endoscopic image (see Table 2), the model in this paper also shows good performance. By calculating the K-ACC metric, it can be found that the model in this paper has the best performance in text output description, except for one disease type, Glycogenic Acanthosis, outperforming by 20.2% for Overall. In summary, compared with other SOTA AI models, the model described in this paper has significant advantages in both diagnostic performance and text output performance for description. Notably, during inference on the test set, the model relies solely on endoscopic images as input, without any accompanying textual information.

To better demonstrate the diagnostic efficacy of our model, a human-computer comparison was performed. As shown in Table 3, the ACC metric of the model proposed in this paper is the highest, whether for WLE or NBI. For WLE, it is at least 2.14% higher; for NBI, it is at least 1.39% higher. This indicates that the overall diagnostic performance of the proposed model for the four diseases is the best. When it comes to the diagnosis of esophageal cancer, a key disease, we can observe that under WLE, although the PREC metric of the proposed model is the lowest, the REC metric is the highest. This implies that the diagnostic strategy of the proposed model under WLE is not conservative, resulting in a lower rate of missed diagnoses than that of doctors. Under NBI, both the PREC and REC metrics of the proposed model are the highest, which means that the proposed model can achieve both accurate prediction and comprehensive coverage under NBI, leading to low rates of both missed and mistaken diagnoses. On the contrary, looking at the diagnostic result metrics of endoscopists in the competition under WLE and NBI, except for Senior1, the other three endoscopists have the same phenomenon: a relatively high PREC metric and a relatively low REC metric (the maximum difference between the two metrics is over 40%). This shows that endoscopists’ diagnostic strategies tend to be conservative. Although the misdiagnosis rate is reduced, the missed-diagnosis rate is increased. This phenomenon poses a significant risk to patients during the screening process.

Our study was designed to explore the potential of pre-trained VL models in accurately diagnosing esophageal whitish plaques. The results of our research suggest that the proposed model demonstrates high diagnostic performance, which can effectively reduce the diagnostic errors made by endoscopists. It is anticipated that this model will offer real-time guidance for medical decision - making, minimize the necessity for excessive biopsies, and ultimately curtail medical resource expenditures. Nonetheless, our study was not without limitations. First, the classification of esophageal whitish plaques was only applicable to WLE and NBI images, excluding endoscopic videos. Second, due to the limited availability of data, this study only concentrated on four common diseases, overlooking other conditions presenting with esophageal whitish plaques, such as esophageal ectopic sebaceous glands, esophageal xanthomas, and esophageal granular cell tumors. Third, as this was a single-center retrospective study, the performance of the model requires further validation through multi-center studies. Finally, except for early esophageal cancer, which was confirmed by histopathology, the ground-truth labels for fungal esophagitis, esophageal papilloma, and glycogenic acanthosis were based solely on endoscopic diagnosis by experienced clinicians, without histopathological confirmation.

Future work should prioritize three key directions to build upon this research. First, there is a critical need to develop and validate real-time video diagnostic models to transition this technology from static image analysis to dynamic clinical use during live endoscopy. Second, the AI system’s classification capabilities must be expanded to include a broader differential diagnosis of esophageal whitish plaques, such as ectopic sebaceous glands, xanthomas, and granular cell tumors, to improve its clinical utility and reduce misdiagnosis. Finally, the model’s generalizability and robustness must be rigorously evaluated through large-scale, multi-center prospective studies to ensure its efficacy across diverse patient populations and clinical settings. The collection of a large amount of multi-center data is very necessary, and the annotation of the data not only includes class information but also text description information.

## Methods

This study was conducted in accordance with the Declaration of Helsinki and was approved by the Ethics Committee of Shandong Provincial Hospital (SWYX: NO.2023-427). The study was retrospective and received approval from the Ethics Committee to waive patients’ informed consent.

### Datasets

In this study, we retrospectively collected 13,922 endoscopic images from 2048 patients from January 2009 to July 2023 at Shandong Provincial Hospital. Esophageal cancer was diagnosed based on pathology, while esophageal papilloma, fungal esophagitis, and glycogenic acanthosis were diagnosed by experienced endoscopists. Poor-quality images, such as those that were blurry, with excessive mucus or food debris, or lacking appropriate inflation, were excluded from the study.

In our retrospective study, all selected endoscopic images include 4433 images from 504 patients with fungal esophagitis, 2963 images from 358 patients with early esophageal cancer, 3383 images from 663 patients with esophageal papillomas, and 3143 images from 523 patients with glycogenic acanthosis. Each disease contained both WLE and NBI imaging modalities. The images of fungal esophagitis, early esophageal cancer, esophageal papilloma, and glycogenic acanthosis were randomly selected and divided into a training set and a test set, with 70% of the images in the training set and 30% in the test set In the experiments, random selection is conducted three times, and the experimental results are averaged based on these three selections. The annotations for all images in both the training set and the test set comprise not only category labels but also descriptive textual descriptions of the corresponding images. The textual description for each endoscopic image mainly consists of three parts: location, appearance description, and disease name. For example, ”This image shows the esophagus. There are whitish plaques on the inner wall, and it is most likely Esophageal Cancer.”

### Disease Diagnostics and Description on BLIP

**Fig. 2:**
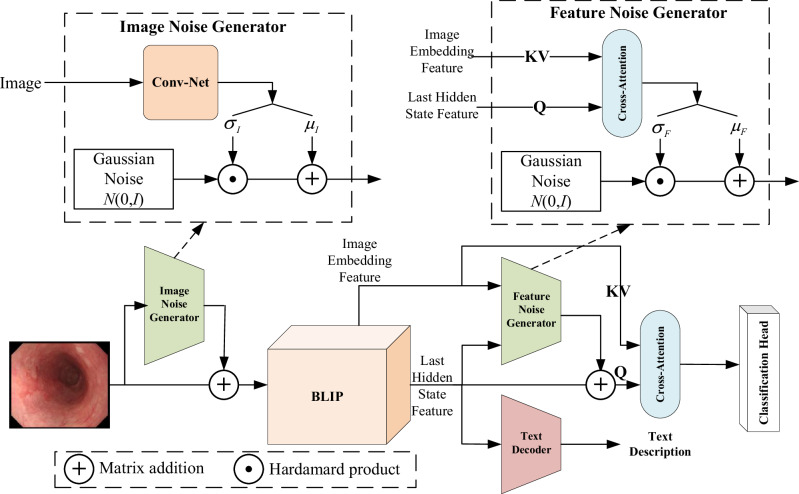
The framework of our proposed system, which includes two important modules for adding positive-incentive noise. Image Noise Generator, and Feature Noise Generator.

Traditional CAD systems for digestive endoscopy images based on deep learning typically reduce the diagnostic process into an image classification task, which only outputs categorical labels (disease types) without supplementary descriptive information such as imaging modality, anatomical location, symptoms, or disease progression. Descriptive information, inherently in natural language form, exhibits a significant modality discrepancy with the input endoscopic images. Cross-modal information output has long been a challenging direction in deep learning; however, recent advancements in large VL model research have enabled notable breakthroughs. Leveraging the cross-modal output capability of large VL models, this study proposes a novel digestive endoscopy CAD system that simultaneously generates diagnostic predictions and descriptive annotations (as shown in Fig. 2). The proposed system is based on the BLIP network architecture, incorporating an input-stage forward noise generator designed to enhance the endoscopic image data through positive-incentive noise injection. At the output-stage, alongside standard text generation capabilities, a positive-incentive noise generator is also applied to the last hidden state features of the BLIP network. These enhanced features are then fused with the original image embeddings of BLIP via a cross-attention module before being fed into a dedicated classification head for diagnostic outputs. The following sections will provide a detailed introduction to this framework.

#### Image Noise Generator

As described in Section 4.1, the collected image dataset in this study comprises fewer than 15,000 samples, which is insufficient for training large VL models. Even when employing the fine-tuning, such limited data volume struggles to guarantee satisfactory training outcomes. Inspired by positive-incentive noise techniques,we added positive incentive noise to both the input (or output) end of the BLIP model. This noise is generated driven by the image input (or image embedding features). According to the theory of^23^, this positive incentive noise is of the same origin as the image signal, and there is mutual information between them. It can effectively reduce the entropy of the classification task, thereby improving the performance of the classification system. This work introduces an image noise generator module at the input stage of the BLIP backbone network (Fig. 2 left). The architecture of this generator is straightforward: it takes an image tensor as input and processes it through a convolutional neural network (Conv-Net) to output two scalar values, *σ*_*I*_ and *μ*_*I*_. If the input image is denoted as $I\in {{\mathbb{R}}}^{m\times n}$, we have $\left({\mu }_{I},{\sigma }_{I}\right)={f}_{\alpha }\left(Conv-Net\left(I\right)\right)$, where the function *f*_*α*_ can be implemented by using various neural network architectures.

Here, *σ*_*I*_ serves as the variance parameter and *μ*_*I*_ as the mean parameter for the generated noise. These scalars are combined with a Gaussian noise distribution N(0, 1) via Hadamard product and matrix multiplication operations, producing a noise image matching the original input dimensions. This additive noise image is then superimposed onto the original image before feeding into the BLIP backbone. The Conv-Net architecture can be dimensionally adapted depending on hardware/software constraints to achieve optimal computational efficiency.

#### Feature noise generator

The noisy image tensor is fed into the BLIP backbone network, where the Image Encoder module generates the Image Embedding Feature, while the final hidden layer of the BLIP model yields the Last Hidden State Feature. These two feature maps incorporate information from both visual and textual modalities, remaining mutually correlated despite their distinct modalities. They serve as inputs to drive the classifier’s diagnostic output. To enhance classification performance, we introduce the positive-incentive noise to the Last Hidden State Feature, generated by the Feature Noise Generator module (as shown in the right part of Fig. 2). This module takes the Image Embedding Feature and Last Hidden State Feature as inputs, which are processed through a cross-attention mechanism to compute the mean *μ*_*F*_ and variance *σ*_*F*_. They are then fused with a Gaussian distribution N(0, 1) to produce the desired positive-incentive noise. If the Image Embedding Feature is denoted as ${I}_{e}\in {{\mathbb{R}}}^{d}$,the Last Hidden State Feature is denoted as $L\in {{\mathbb{R}}}^{d}$, the cross-attention module is denoted as

1 $$CrosAtten\left(Q,K,V\right)=Sof{t}_{MAX}\left(\frac{Q{K}^{T}}{\sqrt{d}}\right)V$$

then we have $\left({\mu }_{F},{\sigma }_{F}\right)={f}_{\theta }\left(CrosAtten\left(Q=L,K={I}_{e},V={I}_{e}\right)\right)$,where the function *f*_*θ*_ can be implemented by using various neural network architectures.

#### Loss function of training

The proposed system comprises two output modules: the classification head module and the text decoder module. The text decoder module, inherited from the original BLIP architecture, generates image descriptions and outputs the built-in text loss function of the BLIP model, denoted as. The classification head module is configured as a stacked structure (CHNet) consisting of convolutional layers, ReLU layers, pooling layers, and fully connected layers. Its output passes through a Sigmoid function to produce the predicted diagnostic logits

2 $${P}_{l}=Sigmoid\left(CHNet\left(CrosAtten\left(Q={L}_{N},K={I}_{e},V={I}_{e}\right)\right)\right)$$

where *L*_*N*_ represents the noise-injected Last Hidden State Feature. During training, the classification head’s loss function is defined as:

3 $${\ell }_{c}=CrossEntropy\left({P}_{l},{Y}_{l}\right)$$

where *Y*_*l*_ denotes the ground truth of classification outcomes. Consequently, the overall loss function of the entire system is formulated as:

4 $$\ell ={\lambda }_{1}{\ell }_{t}+{\lambda }_{2}{\ell }_{c}$$

where *λ*_1_ and *λ*_2_ are the weights of text loss and classification loss, and *λ*_1_ + *λ*_2_ = 1. The text loss *ℓ*_*t*_ corresponds to the original BLIP model’s built-in text loss, which is directly used in this study without modification.

#### Fine-tuning of pre-trained BLIP

The BLIP model used in this paper is a pre-trained model. To enable the pre-trained BLIP model to have a good performance on our task, we employ the LoRA (Low-Rank Adaptation)^24^ method to fine-tune large-scale pre-trained models, aiming to enhance their performance on the diagnosis and description of digestive endoscopy images, while reducing the number of parameters and training overhead. We apply LoRA to the ”query” and ”value” modules of the pre-trained BLIP model. These modules play a crucial role in the self-attention mechanism. By introducing low-rank matrices into these modules, we effectively enhance the model’s performance on specific tasks. Fine-tuning these target modules allows the model to retain pre-trained knowledge while quickly adapting to new tasks of diagnosis.

## Data Availability

The datasets generated and/or analyzed during the current study are not publicly available due to patient privacy and institutional policy restrictions. However, all reasonable requests for academic use of the preprocessed in-house and analyzed data can be addressed to the corresponding author.

## Code availability

The code used in this study is not publicly available due to copyright restrictions.
